# Supplementary material for: Are carbon emissions trading and green financial instruments synergistic? -Comprehensive quantitative research based on content analysis
Source: PLoS One. 2024 Mar 7;19(3):e0298601. doi: 10.1371/journal.pone.0298601 (PMC10919601; doi:10.1371/journal.pone.0298601)
Supplement: S1 Data — (ZIP) [file pone.0298601.s001.zip › Data/地级市Z_F工作报告.zip!/╔╜╢1⁄2/╝├─╧╩╨/└┤╫╘╣1⁄2╓┌║┼í╛┬φ┐╦╩2╛▌═°í┐_2005.docx]

各位代表：

　　现在，我代表市人民政府向大会报告工作，请予审议，并请各位政协委员和其他列席人员提出意见。

　　一、2004年全市经济和社会发展取得显着成效

　　过去的一年，全市人民以党的十六大和十六届三中、四中全会精神为指导，认真落实科学发展观，积极贯彻宏观调控政策，紧紧围绕“发挥省城优势、发展省会经济”总体思路，着力解决经济社会发展中的突出矛盾和事关人民群众切身利益的突出问题，解放思想、干事创业，深化改革、突破难点，开拓进取、扎实工作，圆满完成市十三届人大二次会议确定的各项主要任务。

　　国民经济快速协调发展。牢牢把握发展第一要务，努力转变经济增长方式，不断加大结构调整力度，推动经济快速协调发展。经济发展速度加快。初步核算，全市实现生产总值1618.9亿元，增长15.6%，人均3338.6美元。其中，一产118.7亿元，二产742.4亿元，三产757.8亿元，分别增长7.8%、19.8%、13%。全市境内财政总收入377.8亿元，增长18.9%，其中地方财政一般预算收入89.04亿元，可比增长20.9%。全社会固定资产投资651.3亿元，增长29%。社会消费品零售总额621亿元，增长15.6%。各项存款余额2991亿元、各项贷款余额2830亿元，分别比年初增长22.1%和10.8%。经济运行质量提高。规模以上工业利润增长45.1%，继续呈现利润增幅高于销售收入增幅的良好态势。工业经济效益综合指数194.4%，上升28.8个百分点。销售收入过亿元工业企业232个，增加63个，其中过100亿元的3个。新建续建亿元以上项目274个，对经济发展的支撑带动作用明显提高。经济内部结构优化。三次产业比重由7.6：44.6：47.8调整为7.3：45.9：46.8，二产提高1.3个百分点。固定资产投资率达到40.2%，提高3.2个百分点。非公有制经济比重达到37.3%，提高1.9个百分点。高新技术产值比重达到28%，提高2个百分点。经济外向度15.6%，提高3.4个百分点。实际利用外资(新口径)3.17亿美元，增长21.5%；进出口总额30.5亿美元，外贸出口13.7亿美元，分别增长51.2%和64.3%，实现了历史性突破。

　　“三农”形势发生积极变化。把破解“三农”问题作为全市工作重中之重，制定实施了“6521”和“1020”工程，积极探索以工促农、以城带乡反哺农业新机制。粮食产量明显增长。确定了26.7万公顷基本农田、稳定粮食总产210万吨以上、 地方储备粮24.5万吨的“三条底线”，实现粮食总产242.7万吨，增长10.1%，达到近五年最高水平。农民收入大幅增加。全面落实增收措施，加大农业结构调整力度，附加值较高的蔬菜、畜牧、林业产值占农业总产值的比重分别达到30.3%、35.2%和2.2%；加快发展农村二三产业，加速转移富余劳动力，强化各项支农政策，农民人均纯收入达到4116元，增长13.7%，8年来首次超过城镇居民收入增幅。农民负担切实减轻。4369万元粮食直补资金全部发放到农民手中，章丘、市中、历下、槐荫和高新区提前三年免征农业税，其他县区减征3个百分点，全市农民减负1.58亿元，人均46元。扶贫脱困步伐加快。强化“一县十三乡”脱贫工作，有7.8万人脱贫，21.7万贫困人口人均增收400元。生产条件改善较大。市财政支农投入5.25亿元，同比增长46%。新建改建乡村水泥(沥青)路2056公里，连通750个村庄，受益人口50万，村村通客车率达到99.1%。新增和改善灌溉面积分别达3000公顷和1.3万公顷，新增蓄水能力240万立方米，解决了28.9万人的饮水困难。

　　城市建设实现新的突破。按照“东拓、西进、南控、北跨、中疏”空间战略和“新区开发、老城提升、两翼展开、整体推进”基本思路，坚持“三高”方针，精心组织策划，完善运行机制，大力推进城市建设。城市规划全面展开。高起点搞好城市总体规划修编，完成经济社会发展、人口和用地、道路交通、市政设施、生态环境5个大类36个专题研究项目，形成《济南市城市总体规划》纲要，通过专家论证和市规划委员会审查；组织编制了东部新城中心区规划设计方案、城市综合交通以及25条道路等重点详规编制和重点片区空间布局规划。重点工程进展顺利。“十大工程”取得阶段性成果，累计开工项目209个，竣工112个，当年完成投资201亿元，是我市城建史上投入最多、变化最大的一年。新建改建市区道路43条，总长度260公里，铺装各类管线400公里，整治疏浚河道70公里，开工住宅面积320万平方米，新增绿化面积478.4万平方米，城市绿化覆盖率39.1%；经十路、经一路、济泺路、工业南路等一批重要干道和国际机场航站区、燕山健身广场、植物园、体育中心等一批重点工程相继竣工，城市面貌局部发生质的变化。综合整治初见成效。投入1.9亿元对985条背街小巷进行综合整治，集中治理占道经营、大气扬尘、乱倒渣土等突出问题，显着改善了城区居民生活环境；加大封井保泉措施，实现群泉全年喷涌；电力、通讯等基础设施条件进一步改善，电网建设成效显着，实现了“零拉路”运行，我市成为全国供电环境最好的省会城市之一。

　　落实宏观调控政策取得成效。全面正确理解和落实中央宏观调控政策，紧紧把握宏观调控机遇，抓住影响经济发展的突出问题，集中开展专项治理整顿，努力化解经济运行中不健康、不稳定因素，经济发展秩序进一步规范。土地市场秩序明显好转。以保护基本农田、加强土地用途管制、推进市场化供地为重点，深入整顿土地市场秩序，依法查处各类土地违法案件，集中处理土地历史遗留问题，全面加强土地市场建设和保护土地资源各项措施，基本解决了土地市场存在的突出矛盾和问题，推动管地用地秩序纳入依法规范的轨道，市、县政府当年实现土地纯收益超过前5年总和。开发区整顿成果显着。以加强用地管理、提高土地使用效率为重点，共清理各类开发区52个，撤销40个，核减规划面积3.5万公顷，保留开发区12个，规划面积1.1万公顷，促进园区合理布局和集约发展。在建项目清理成效明显。停建和取消立项项目13个，整改项目27个，促进了投资方向和结构的优化；加强经济运行薄弱环节，煤电油运正常供应，保障了生产生活需要。建设市场秩序逐步规范。以加强规划管理为重点，严厉查处违法建筑和违规建设行为，健全建设项目动态巡查制度，有效规范了建设行为。建章立制取得成效。出台规范性文件88件，建立起规范有序的发展和建设新秩序，特别是完善征地、拆迁政策，有效维护了被征地、拆迁群众切身利益。

　　各项改革继续深入推进。以转变政府职能为核心，以优化发展环境为重点，相继推出一系列改革措施。政府机构改革成效明显。加强社会管理和公共服务机构，精简职能错位、交叉的政府机构，增减相抵净减机构17个，精简幅度18%。行政审批改革深入推进。按照行政许可法要求，审查修改政府规章及规范性文件73件，清理行政许可事项450项，清理行政许可主体27个，清理收费项目75项，实现了行政许可职权法定。进一步强化审批中心职能，精简审批程序和环节，审批效率明显提高。政务信息公开全面推行。建立政务信息公开和政府重大决策事项听证制度，完善人大代表、政协委员建议和提案征询办理制度，健全了政务信息公开各项制度，提高了政府决策的民主性、科学性和透明度。财税体制改革稳步推进。建立税收属地管理、主体税种两级分享的财政管理体制，理顺市与县(市)区财政分配关系，解决了收入混级混库、级次间挤挖等问题，促进了资源市场化流动和财政增收。国资管理体制改革持续深化。深入推进重点企业和困难企业产权制度改革，以前所未有的力度推动ST上市公司整合重组、走出困境，上市公司积淀的矛盾和问题基本得到化解；组建新的国有资产管理委员会，打通非国有资本进入国有经济和劣势国有企业退出两大通道，规模以上工业国有经济比重下降3.3个百分点。

　　社会事业有了新的发展。坚持统筹经济与社会协调发展，大力推进科教兴市、人才强市战略，逐步加大社会事业投入，各项社会事业加快发展。科技实力显着增强。加快科技创新体系建设，积极推进产学研结合和高新技术产业发展，科技创新和成果转化成效明显，全市共取得重要科技成果90项，获得国家、省以上科技奖励25项；建成省级以上创业服务中心4家，生产力促进中心5家，高新技术开发区的技术创新、企业孵化和高新技术产业化优势日益突出。教育事业全面发展。优质教育资源显着增加，普通高中课程改革实验全面展开，高中阶段教育普及率达到85%，国民教育、职业教育、成人教育和农村教育逐步适应城市定位和产业升级需要。人才队伍建设成效显着。来济创业吸引力增强，专业技术人才总量达到59万人，同比增长6%。文化事业不断发展。文化体制改革不断深化，文化市场健康有序，社区文化丰富多彩，艺术创作成绩显着，获省级以上奖励281项。体育事业加快发展。成功举办了2004年亚洲杯足球赛和世界摩托艇锦标赛等大型赛事，竞技体育获省级以上金牌134枚，实现了国际残奥会金牌零的突破。公共卫生保障有力。以疾病预防控制和农村公共卫生为重点，全面加强“两个体系”建设，应对公共卫生突发事件能力明显增强。计划生育稳步推进，人口自然增长率控制在3.8‰。创建文明城市扎实有效。深入开展精神文明建设活动，“迎亚足赛·创文明城”主题行动结出丰硕成果，为民办百件实事全部达到预期目标。新闻出版、广播电视、社会科学、档案史志、外事侨务、民族宗教、妇女儿童、老龄、残疾人等事业都有了新的发展。

　　人民生活水平进一步提高。坚持以人为本的执政理念，切实维护人民群众切身利益，集中抓好扩大就业和社会保障工作，人民生活进一步改善。就业再就业工作加强。各项政策进一步落实，工作机制不断强化，服务网络更加完善，通过各种途径安置就业再就业11.88万人，其中安置困难群体1.12万人，城镇登记失业率为3.89%。城市社会保障能力继续提高。加强责任、政策、社区、服务、监督五个体系建设，健全基金预算管理、征缴清欠、激励约束和诚信监督四项制度，基本养老保险、基本医疗保险、失业保险参保人数和享受城镇最低生活保障人数分别为93.5万、53.1万、62.5万和6.56万人。社会救助体系初步形成，社会福利设施进一步改善，农村最低生活保障制度逐步规范。转移农村富余劳动力14.7万人，清欠农民工工资4.9亿元。城乡居民收入进一步提高。城镇居民人均可支配收入10798元，增长10.2%；城乡居民储蓄余额870.5亿元，比年初增长14.8%。城乡消费水平进一步提升。城镇居民人均消费性支出8471元，同比增长6.2%，居民消费价格指数为102.5%。住房公积金制度推行力度加大，作用日益突出。城市居民人均住宅使用面积19.5平方米，农村居民人均生活用房面积32.9平方米，分别增加0.65和0.4平方米。城市供热普及率43%，城市用气普及率98%，分别增长1和0.6个百分点；每百户居民拥有私人汽车3.67辆、电脑49台、移动电话129.7部，分别增长22.3%、6.5%和17.5%。

　　“平安济南”建设不断加强。坚持依法治市，强化综合整治措施，推动工作重心下移，着力打造全省“首善之区”。社会持续稳定安全。扎实开展居民社区、公共复杂场所、驻地单位“基层创安”活动，初步形成全方位的平安城市建设格局。深入开展“严打”斗争，重点整治突出治安问题，推进治安防范机制市场化，治安问题和刑事案件明显减少，人民群众安全感普遍增强。积极预防、疏导和化解社会矛盾，加强基层基础工作，围绕重点工程拆迁建设、农村税费改革、企业改制重组等热点难点问题，加强矛盾纠纷排查调处，不稳定隐患逐步减少。信访工作继续加强，大量信访问题得到化解。安全生产专项整治成效明显，安全事故发生起数和伤亡人数大幅下降。双拥共建深入开展，获得全国双拥模范城“五连冠”，国防教育、征兵工作、国防后备力量和人民防空建设不断加强。政府依法行政水平进一步提高。各级政府按照为民、务实、清廉的要求，不断改进工作作风，加强自身建设，更加自觉地接受同级人民代表大会及其常委会的监督，更加注重加强同人民政协的联系，高度重视人大代表建议和政协提案办理工作，全年办理人大代表建议290件、政协提案459件。注重从源头上预防和治理腐败，廉政建设和反腐败斗争逐步深入，严肃查处各类违法违纪案件，纠正部门和行业不正之风，从体制、机制上加强审计监督，推动了政府自身建设和作风转变。

　　各位代表，过去一年济南的发展和进步，是省委、省政府和市委正确领导的结果，是全市人民开拓进取、努力奋斗的结果，是人大、政协、各民主党派监督支持、戮力协作的结果，是驻济部队、各界人士积极参与的结果。在此，我代表市政府向辛勤工作在各行各业的全体市民，向给予政府工作大力支持的各级人大代表、政协委员、各民主党派、工商联、各界人士，向关心帮助地方工作的驻济人民解放军、武警官兵，向为济南发展做出贡献的港澳台同胞、海外侨胞、国内外朋友们表示崇高的敬意和衷心的感谢！

　　各位代表，过去一年取得的成绩令人鼓舞，但前进道路上还有不少矛盾和问题。这主要是：深层次结构性矛盾比较突出，全市工业经济、非公有制经济、县域经济、固定资产投资的比重和经济外向度，虽然有不同程度的提高，但仍低于全省平均水平，这些结构性的差距，直接影响到经济发展的速度、活力和后劲；农民持续增收的基础不稳固，去年我市农民收入增长较快，其中价格因素和政策因素影响较大，县域二三产业总量小，劳务输出成效不明显的状况不改变，就难以支撑农民收入持续快速增长；转变经济增长方式步伐不快，去年主要原材料、燃料和动力价格上涨，导致工业企业亏损面、亏损额较大幅度上升，今年主要原材料价格上涨局面还将持续，加快增长方式转变、推进循环经济发展、消化增支减利因素十分迫切；社会保障体系建设相对滞后，随着各项改革逐步深入，经济结构调整力度加大，大量城镇失业人员需要安置，农村富余劳动力需要转移，城乡困难群体需要救助，迫切需要保障类别逐步增加、保障范围逐步扩大、保障标准逐步提高，健全和完善社会保障体系的任务还相当繁重；发展环境问题仍然比较突出，服务意识差、办事效率低和执法不规范的现象还不同程度存在，对“三乱”问题、行业不正之风和干部腐败现象社会反映还比较强烈，各级政府转变职能、改进作风、依法行政仍需做出更大努力。我们必须正视这些突出矛盾和问题，立足当前，着眼长远，多措并举，标本兼治，坚持用改革的办法，从体制上、机制上、政策上逐步加以解决。

　　二、2005年经济社会发展的主要任务和工作重点

　　今年是贯彻落实科学发展观、巩固宏观调控成果、保持经济社会良好发展态势的重要一年，也是全面实现“十五”目标、为“十一五”发展奠定坚实基础的关键一年。在新的一年里，政府工作的指导思想是：坚持以邓小平理论和“三个代表”重要思想为指导，认真贯彻党的十六大、十六届三中四中全会和中央、全省经济工作会议精神，牢牢抓住战略机遇期，全面落实科学发展观，始终坚持以人为本，充分发挥省城优势，大力发展省会经济，把握“三个关键”、落实“三个注重”、实现“三增目标”、实施“六个突破”，推动全市经济社会更快更好发展。

　　——把握“三个关键”。加快经济结构调整、推进经济体制改革、转变经济增长方式，是做好今年及今后一个时期经济工作的着力点，是解决经济社会发展深层次矛盾的关键。要充分认识把握“三个关键”的重要性、长期性和艰巨性，抓住制约发展的结构性矛盾和问题，着力深化经济体制改革，推进经济增长方式转变。

　　——落实“三个注重”。更加注重解决经济社会发展中的突出矛盾、更加注重解决关系群众利益的突出问题、更加注重处理好改革发展稳定的关系，是构建社会主义和谐社会必须着重把握好的重大问题。落实“三个注重”，关键是把以人为本的执政理念贯穿到经济社会发展全局，落实到工作的各个方面，把改革的力度、发展的速度与人民群众的承受程度统一起来。

　　——实现“三增目标”。实现地区经济总量明显增长、综合经济效益明显增加、经济发展后劲明显增强，是检验今年经济工作成效最重要的三大目标。生产总值是反映地区实力的核心指标，财政收入是反映经济效益的综合指标，全社会固定资产投资是反映发展后劲的重要指标。这三大指标在确保完成预期目标的基础上，实际工作中要力争完成得更好一些。

　　——实施“六个突破”。在调整经济结构、解决“三农”问题、统筹区域发展、引资创汇、建设生态城市、加大社会投入等六个方面实施重点突破，是全面落实科学发展观、统领经济社会发展全局的重大思路。围绕实施“六个突破”做好各方面工作，不仅对于实现今年经济目标非常必要，而且对经济社会快速、协调、可持续发展至关重要。

　　全市经济社会发展的主要预期指标是：生产总值增长14%；地方财政一般预算收入增长15%；全社会固定资产投资增长30%；社会消费品零售总额增长15%；外贸进出口增长20%；实际利用外资增长80%；城镇居民人均可支配收入增长8%；农民人均纯收入增长8%；城镇登记失业率控制在4.2%以内，人口自然增长率控制在5.9‰以内。

　　2005年经济社会发展的重点是：

　　(一)在经济结构调整上重点突破，加快构筑现代产业体系。把调整优化经济结构、加强薄弱环节作为长期战略任务，全力推进，重点突破。大力推进工业经济加快发展。坚持新型工业强市战略。以信息化带动工业化，走科技含量高、经济效益好、资源消耗低、环境污染少、人力资源得到充分发挥的新型工业化道路。加大“2133”工程实施力度。力争规模以上工业销售收入突破2000亿元，工业投入突破350亿元，规模以上工业企业突破2000个，销售收入过亿元工业企业突破270个。强化“三大群体”带动作用。强化重点园区带动作用，整合优势园区，集中连片发展，实现设施建设、项目落地、项目投产的重大突破，形成新型工业产业集聚区；强化优势产业带动作用，以龙头企业优势产品为依托，以配套企业合理布局为基础，以上下游产品有机链接为纽带，加快构筑产业链条，先期实施钢铁、汽车、电子、石化四大产业链，创造条件培育新的产业链；强化骨干企业带动作用，以主业突出、业内领先的企业为重点，以资产重组为手段，加大扶持力度，培植一批具有驰名商标、名牌产品的大型骨干企业，力争销售收入过10亿元企业达到20家。加快增长方式转变。开发与节约并举，发展循环经济，以资源依赖性行业为重点，推进节能降耗、洁净生产，提高资源利用率。积极推进建筑业壮大发展。按照壮大实力、增强活力、增加效益的思路，加快改制重组步伐，积极发展对外承包，规范建设市场秩序，形成一批富有竞争力的建筑企业集团。大力推进现代服务业加快发展。坚持三产富市，把发展服务业摆在“发挥省城优势，发展省会经济”的突出位置，扩大开放，提升层次，重点推动物流、会展、旅游、金融等尽快形成支柱产业。大力发展现代物流业。加快物流平台建设，发展大型物流基地、名牌企业和第三方物流，构建现代化区域性物流枢纽。大力发展会展旅游业。努力打造特色突出、规模较大、效益显着的会展品牌，重点办好一批大型展会。发展大旅游，开拓大市场，构筑大产业，全面提升行、住、吃、游、购、娱六要素，全力打造泉城旅游品牌，重点发展国际旅游，力争展会总数、旅游人数、旅游收入实现大的突破。加快发展金融业。推进区域金融中心建设，引进境内外金融企业，积极培育保险市场，加大上市公司重组和后备上市资源培植力度，强化金融业对经济社会发展的带动和服务功能。大力发展中介服务业。着力培育和发展金融担保、信用评价、资产评估、公证仲裁等各类中介机构和组织。加快提升传统服务业。以促进流通现代化为目标，拓展连锁经营、物流配送、电子商务等现代营销方式。修编完善城市商业网点规划，引导商业网点合理布局，规范发展，加快便民市场建设，方便群众生活。大力推进高新技术产业加快发展。突出抓好高新技术产品、产业和园区，高新技术产值比重达到30%以上。优先发展电子信息、生物制药、新材料等高新技术产业，形成一批具有国内领先水平的技术与产品，引进和培育一批具有国际市场竞争力的高新技术企业，加快以高新区为龙头的各类科技园区建设。推进高新技术和先进适用技术改造提升传统产业，以信息化为主要手段，实施一批以装备制造业、农业和商贸流通业为重点的示范工程。加大技术创新投入，建立以企业为主体、以知识产权为核心的技术创新体系和市场化成果转换机制，建好各级技术中心等创新平台，提高技术含量和自主创新能力。大力推进非公有制经济加快发展。加快所有制结构的调整优化，非公有制经济增加值比重达到40%以上。放手发展非公有制经济，坚持政策启动、改革推动、骨干带动、科技拉动“四动并举”方针，鼓励、引导民间资本投向主导产业，推动民营企业参与国有企业重组改造和上市融资，扶持优势民营企业做大做强，吸引外地民营资本来济投资兴业。加快建立促进发展服务体系。健全民营经济融资担保、信用评价、人才培训、技术信息、法律服务和行业协会六大服务体系，用好中小企业发展专项资金，重点支持融资担保、创业资助和市场开拓。

　　(二)在解决“三农”问题上重点突破，促进城乡经济发展良性互动。毫不动摇地把解决“三农”问题摆在重中之重，以促进农民增收为核心，加快完善以工促农、以城带乡反哺农业机制，提高农业综合生产能力和市场竞争力。加大农民增收力度。继续实施“6521”工程，全面落实增收途径和各项措施。把加大农村劳动力转移作为促进农民增收的重要手段，加快小城镇建设和二三产业发展，把握市场需求，定向开展技能培训，健全劳动力资源、劳动力市场需求信息库和农村劳动力转移服务网络，形成市场订单—技能培训—输出服务体系，培训10万人，转移14万人。加大结构调整力度。积极促进农业内部结构调整优化。在确保粮食总产210万吨的基础上，放开手脚扩大畜牧、蔬菜、林果和渔业等高效农业生产，加大科技兴农工作力度，加快农业产业化、标准化、国际化进程。大力推进“双十工程”，扶持一批竞争力强、带动力大的龙头企业。积极发展现代都市农业。以服务城市为目标，以市场需求为导向，积极发展休闲、观光、生态农业。加大支农投入力度。大力实施“1020”工程，确保各级财政支农投入增幅高于财政收入增幅。加强以农田水利为重点的基础设施建设，实施30座小型水库除险加固和村村通自来水工程，实现358个村、24万人饮水安全，基本完成村村通油路工程。加快农村教育、卫生、文化等公益设施建设，完成农村校舍危房改造工程，加快村村通有线电视建设。按照“产业扶贫、到村到户”的思路，以“一县十三乡”为重点，实现9万人脱贫。加大政策落实力度。切实减轻农民负担，进一步落实各项减征措施，全部免征农业税及附加，严格落实粮食直补、良种补贴和购置大型农机具补贴政策，确保全部补贴到位。完善农村土地承包制度，规范农村土地承包经营权流转程序和行为，切实维护农民的合法权益。

　　(三)在统筹区域发展上重点突破，促进全市经济协调发展。加快解决区域经济发展不平衡的矛盾，是增强我市整体实力的迫切需要和加快发展的潜力所在。必须统筹规划，分类指导，优势互补，共同发展。提升中心区。针对土地资源紧缺、发展空间狭小的突出矛盾，历下、市中、槐荫、天桥立足各自实际，发挥驻地优势，整合城区资源，发展省会经济。突出发展总部经济。吸引各类投资管理机构、国内外知名企业总部及其分支机构来济落户，充分发挥总部经济的聚集带动作用。突出发展楼宇经济。以区位优势为基础，以高档商务楼为载体，科学定位，综合配套，整合一批、新建一批、打造一批品牌商住楼宇，引进一批专业性、集聚性、拉动性强，产出高的管理型和智能化高科技企业及现代服务企业，形成新的经济效益高地。突出发展特色经济。加快培育一批商贸、科技、文化、娱乐、餐饮、民俗特色专业街和片区。做强近郊区。章丘、历城和长清实行工业化和城市化有机结合，突出自身特色，加快与市区对接。拉开发展框架。统一规划设计，组团式块状开发，建设高水平专业园区。搞好综合配套。加快基础设施建设，增强园区承载能力，营造产业集聚效应。切实加大投入。大力招商引资，突出大项目建设，狠抓大企业膨胀。加强产业发展规划，围绕主导产品，发展配套产业集群。发挥历史文化资源优势，因地制宜发展优势特色产业，全面实现跨越式发展。突破远郊区。济阳、商河、平阴充分发挥土地、劳动力等资源优势，大力引进各种生产要素，因地制宜发展比较优势产业。加快“一跨两接三转移”步伐。加快北跨黄河发展。推进黄河北工业新区建设，以工业“北上”带动城市“北跨”。强化交通衔接，做好产业承接。加快交通支撑体系建设，以高速公路、国省道、村村通、主枢纽场站建设为重点，构筑多元化的快速交通网络；积极承接市区产业延伸，搞好配套加工。加大财政转移支付、农村劳动力转移、工业项目转移力度。远郊三县要把握新的机遇，立足自身努力，发挥本地特色，实现经济发展较大突破。

　　(四)在引资创汇上重点突破，全方位推进对外开放。扭转我市经济外向度低的被动局面，必须在大力调整产业和产品结构的基础上，全力推进招商引资和转变外贸增长方式重点突破。突出重大项目带动。招商引资与城市建设相结合，以城市发展规划为导向，吸引境外资本投资市政公用、基础设施和房地产开发项目；招商引资与国企改制相结合，利用境外上市、企业并购、产权转让、资产置换等多种途径，争取更多外资大项目参与国企改制；招商引资与构筑产业链相结合，重点瞄准世界500强企业和跨国集团，吸引外资丰富扩展产业链；招商引资与园区建设相结合，突出园区带动作用，加强项目落地服务，进一步完善招商引资服务机制，对重大项目实行全程跟踪服务，集中力量突破落地瓶颈，促进重点项目早开工、早投产、早见效。转变外贸增长方式。调整出口结构，集中力量扶持机电产品、成套设备和高科技含量、高附加值产品出口，大力推行国际标准，积极应对技术壁垒，提升出口产品的国际竞争力。进一步落实好出口退税政策，帮助有条件的企业开展外贸经营，壮大出口主体，培植骨干企业，实施多元战略，深度开拓国际市场。加快出口加工区基础设施和“大通关”建设，为外贸出口提供更加便捷的服务。支持企业境外发展。进一步实施“走出去”战略，扩大对外合作，完善境外投资支持和服务体系，支持有条件的企业到境外投资、承包工程，增加劳务输出，扩大发展空间，用好两个市场、两种资源。抓住济南国际机场扩大客货运输能力的机遇，争取多开国际航线，增加航班密度，扩大对外交流。

　　(五)在生态城市建设上重点突破，促进人与自然和谐发展。突出以人为本，坚持“三高”方针，加强城市规划建设管理，打造和谐优美的生态环境。继续完善城市规划体系。以建设生态城市、促进人与自然和谐为目标，全面推进城市总体规划修编，加快分区规划、控制性详规、专项规划等基础规划和乡镇规划修编，重点编制好高新区、经济开发区等重点片区的控制性详规。树立科学规划思想。着眼于建设现代化区域中心城市、提升现代服务功能和城市品位，高起点搞好规划设计，注重生态保护和经济效益，实现功能性、景观性和经济性有机统一。健全规划质量保证机制。开放规划设计市场，引进国内外先进规划设计理念和成果，充分发挥城市规划决策咨询和城建项目集中审批制度的重要作用，提高规划设计和实施水平。加快推进十大工程建设。按照先急后缓、突出重点、标本兼治、集中连片的思路，统筹新区开发与老城提升、统筹城市发展与生态环境，协调推进各项建设。重点搞好城市基础设施建设。围绕实施园区带动战略，在高新技术开发区和经济开发区，加大产业起步区和高校园区的基础设施配套建设力度；围绕重大会展活动和我省争办十一届全运会，集中搞好济南国际会展中心片区配套设施建设，适时启动奥体中心及东部新城建设；围绕保护泉城特色和提升老城品位，重点搞好经十路标准段、大明湖地区、解放阁舜井街片区等综合改造整治；围绕改善城区道路交通，新建改造整修市区40条道路，加快济菏、济莱和大北环高速公路建设；围绕改善群众住房条件，连片建设一批普通商品房和经济适用房，形成方便群众生活的宜居社区。全面提升城市管理水平。按照城市管理重心下移的要求，进一步完善管理运行体制，提高城管综合执法效能。加强环境综合整治。继续实施市容环境综合整治和街巷整治工程，重点整治乱倒渣土、占道经营、施工扬尘、污水排放和乱搭乱建，突出抓好重点道路和重要区域的环境卫生，推进综合整治向城镇延伸，基本完成578条背街小巷整治。完善城市公共服务。充分发挥“12319”热线服务功能，加强城市交通、停车场(点)的管理，优先发展公共交通，切实解决群众关心的热点问题。加强城市生态保护。围绕创建国家环保模范城市和建设生态城市，以改善环境质量、保护群众健康为出发点，以污染物总量控制为主线，提高环境监测能力，从源头上解决污染问题，加大生态环境保护力度，有效防治和减少大气污染、水污染、土壤污染、噪声污染和各种废弃物。全面提高城乡造林绿化水平，新增绿地面积362万平方米，增加生态林6700公顷。继续搞好南部山区水源涵养和治理保护，进一步加大沿黄防风治沙力度。加快分质供水实施步伐，实施严格的地下水管理措施，力争正常水年份泉水常年喷涌。以建设资源节约型社会为目标，重点加强能源、原材料、土地和水资源节约保护工作。

　　(六)在社会事业发展上重点突破，构建良性互动和谐社会。健全多元化社会事业投入机制，保持政府投入稳步增长，引导社会投入持续增加，着力加强社会公共服务投入，促进经济和社会事业协调发展。继续实施科教兴市和人才强市战略。全面加强未成年人思想道德建设，实施农村教育振兴计划，深化基础教育各项改革；面向市场整合职业教育，发展成人教育，规范和促进民办教育，鼓励支持多种模式办学和国际合作办学。加快建设以高等院校和重点科研机构为主体的知识创新体系，促进更多的企业与高校科研机构建立合作关系，争取更多项目列入国家科技计划。加快培育以中介组织为主体的市场化服务体系，推进科技成果向现实生产力转化。抓住培养、吸引、用好人才三个环节，加强人才资源能力建设和高层次人才的培养，积极吸引海内外高级人才，高度重视企业家队伍建设，完善鼓励优秀人才脱颖而出、人尽其才的推进机制。加快推进文体卫等其他各项事业发展。全力打造“文化济南”，继续深化文化体制改革，整合文化资源，发展文化产业，增加对公益性文化事业投入，实施历史文化名城保护工程。努力打造体育强市，深入开展全民健身活动，加大对体育基础设施的投入力度，做好十运会的备战参赛和十一届全运会主会场承办前期准备工作。强化“两个体系”建设，完善公共卫生长效机制和突发事件预警、应急机制，健全社区卫生服务体系和农村新型合作医疗制度；加快卫生资源整合，强化艾滋病等重大传染病防治工作，加快市疾病预防控制中心和市传染病医院建设。创新计生工作运行机制，继续稳定低生育水平，不断促进优生优育。积极做好对台、外事、侨务、民族、宗教、妇女、儿童、老龄、残疾人等工作，推动新闻出版、广播电视、社会科学、档案管理等各项社会事业的发展。深入开展创建文明城市活动。围绕建设“文明济南”，以争创省级文明城市和全国文明城市创建工作先进城市为目标，深入扎实开展文明城市创建活动，坚持经常性工作与集中性行动相结合，制定2005年至2007年创城工作规划，逐级分解目标，落实任务责任，形成全市合力，重点突破与《全国文明城市测评体系》差距较大的薄弱环节，集中抓好群众关注性高、社会影响大、整体带动性强，对推动我市“三个文明”协调发展具有重大作用的主题行动，继续办好广大群众普遍关心的百件实事，弘扬优秀传统文化，传播现代文明知识，促进市民文明素质提高，优化经济与社会发展环境，增强城市的凝聚力和吸引力。继续做好拥军优属工作，认真落实优抚安置政策，大力支持国防建设。

　　(七)强化综合调控手段，增强经济发展活力和后劲。积极推进改革，把握投资力度，努力扩大消费，综合推进经济社会持续健康发展。继续深化各项改革。深化国有企业改革。以建立现代产权制度为重点，打通劣势企业退出通道，推进国有企业股份制改造，企业国有产权比重由51.7%调整到30%。深化投资体制改革。切实落实企业投资自主权，完善政府投资项目决策机制，对非政府投资项目实行核准和备案制。深化财税体制改革。全面落实国库集中支付、政府采购和收支两条线制度，改革政府资金和资产管理制度，清理和剔除不属于公共财政范畴、不符合统筹发展方向的财政支出，解决新财政体制实施中的突出问题；强化协税护税，切实增加财政收入。深化事业单位改革。对行政支持类、社会公益类、经营开发类事业单位，实施不同改革措施，同时推进用人、分配、社会保障等配套改革。深化公用事业改革。进一步放开市政公用市场，鼓励社会和境外资本参与市政公用设施建设和经营。整顿规范市场秩序。完善土地和建设市场管理秩序，规范资本、技术和劳动力市场秩序，大力推进社会信用体系建设。破解土地制约瓶颈。全面准确把握中央宏观调控政策，采取盘活闲置土地、推广多层厂房、非农用地流转、用好产业政策、提高投资强度、争取机动指标、促进村庄整合、开发复垦整理等八条途径，深度挖掘土地潜力，切实解决好用地问题。积极推进经营性用地“招拍挂”，大力提高土地利用效益。保持适度投资规模。保持投资强度。根据结构调整和城市建设发展需要，安排全社会固定资产投资850亿元。着眼于增强发展后劲，规划一批、在建一批、达产一批、储备一批投资几亿、十几亿以上的大高外项目。优化投资结构。重点支持高新技术、高附加值、高出口创汇、高税收产品的投入，统筹安排好基础设施建设和更新改造投入，逐步加大公共产品投入，坚决遏制低水平建设和耗能高、污染大、资源浪费项目。拓宽融资渠道。积极争取政策性资金和信贷资金，大力吸引民间资本、社会资本和境外资本，拓宽市场化、多元化融资渠道。突出消费拉动作用。提高社会支付能力。健全与经济发展相适应的收入增长机制，促进机关事业人员和企业职工收入随着地方财力增长和企业效益提高而增加，增加中低收入群体和特困居民收入，适度调整社会低保和救助标准。发展产业带动消费。增强省会城市集聚功能，大力发展各类消费型产业，扩大城乡居民消费，吸引国内外更多客商来济南消费。加快城镇建设带动消费。加大城市基础设施建设力度，鼓励各类投资主体投资农村商业设施建设，为拓展城乡市场、扩大居民消费创造有利条件。培育热点引导消费。积极培育汽车、住房、电子、通讯产品等新的消费热点，优化消费结构，加强价格调控，改善消费环境，充分发挥消费对经济的拉动作用。

　　(八)坚持以人为本执政理念，确保社会和群众生活稳定。始终把解决好关系广大人民群众切身利益的问题作为政府工作的重点，切实维护群众利益，促进社会公平和稳定。突出抓好就业和再就业。大力开拓就业领域和渠道。把扩大就业和深化改革、调整结构有机结合，落实就业再就业工作责任制，实施创业培训工程，加快劳动力市场建设和社区劳动服务平台建设。完善公益性岗位储备制度。用好用活再就业资金，开发公益性岗位5000个。健全再就业援助制度。大力实施帮扶工程，广泛开展送岗位、送技能、送信息、送服务活动，解决好困难群体再就业问题。全年安置就业再就业12.6万人。不断提高社会保障能力。健全保障机制，强化责任体系，继续巩固“两个确保”和“城市低保”，积极推进“分类施保”，依法加强社会保险费征缴和清欠工作，扩大企业基本养老保险覆盖面。加强农村保险的基础性工作，稳步推进农村社会养老保险工作，完善失业、基本医疗、职工工伤和生育保险制度，完善社会救助和法律援助制度，做好受灾群众生活救济工作。安排2.42亿元，重点支持居民最低生活保障、企业军转干部保障、特困职工救助以及抚恤救灾等社会救助工作。加强劳动保障监察，规范劳动关系，维护职工合法权益。扩大住房公积金覆盖面，提高职工住房保障能力。加强民主法制建设。全面提升依法治市水平。深化“四五”普法教育，做好农村基层换届选举工作，健全完善社区各项民主制度。深入开展“平安济南”建设。全面落实社会治安综合治理各项措施，健全维护稳定、治安的长效机制和经常性严打整治机制，促进基层平安机制建设，提高社会整体防范能力。高度重视人民来信来访工作。完善人民内部矛盾调解工作机制，及时排查调处社会矛盾纠纷，积极预防和妥善处理群体性事件。强化安全生产工作。以防火、防爆、防泄露为重点，推进安全生产质量标准化，严格安全责任制，加大监管力度，搞好专项整治，及时发现和整改隐患，严防重特大事故发生。抓好关系群众切身利益的食品、药品市场专项整治，依法严厉打击制售假冒伪劣商品、走私贩私、非法促销、价格欺诈等违法犯罪活动。

　　三、切实加强政府行政能力和自身素质建设

　　面对新的形势和任务，各级政府和领导干部要坚持以人为本执政理念，全面落实科学发展观，促进政府职能转型，提高科学行政、民主行政和依法行政的能力，加快建设阳光政府、服务政府、法治政府、责任政府，以崭新的面貌和扎实的作风开创经济和社会发展新局面。

　　加快政府职能转变。着眼于释放市场主体的潜力，着力于公共产品的建设，致力于发挥好政府的调控、监管、管理、服务作用，继续用市场的、改革的、创新的办法解决体制机制性问题。深化行政管理体制改革。按照政企分开、政资分开和政事分开的要求，把不该政府管的事，坚决交给企业、市场和中介机构去办，提高行政效能，降低行政成本和商务成本，着力创造公开公正公平的市场环境，切实把政府职能转到经济调节、市场监管、社会管理和公共服务上来。转变政府经济管理方式。提高按市场经济规律办事的意识和能力，提高按国际通行规则办事的意识和能力，提高主要运用经济、法律手段调节经济运行的意识和能力。加强社会管理和公共服务职能。统筹社会事业发展，完善公共服务机制，提高应对公共危机的能力，通过实施有效调控，维护社会公平，使广大群众最大限度享受到改革发展的成果。

　　加强规划管理调控。把编制经济社会发展规划、加强规划调控和管理作为政府的重要职责，以科学发展观统领经济社会发展全局，着眼目标年经济社会发展的客观要求，力求用改革的精神和前瞻的思维，精心搞好各项重大规划的科学论证和社会公示，提高规划的预见性、科学性和指导性，努力把“十一五”规划和2020年经济与社会长远规划编制成为全面贯彻落实科学发展观、切实体现“五个统筹”、推进可持续发展的规划；把城市总体规划和土地利用总体规划修编成为合理开发和有效保护资源、促进城市发展与生态平衡、指导调控经济社会发展和城市建设的规划；把文明城市创建规划修编成为全面推进“三个文明”建设、提升全社会文明素质、推进社会主义和谐社会建设的总抓手。依法维护各项规划的严肃性，严格按照规划的规定和程序开展各项建设，完善动态监管机制，坚决制止随意改变和违反规划的行为。

　　推进政府制度建设。坚持执政为民的根本宗旨，不断提高政府公信力，按照建立公开、廉洁和责任政府的要求，健全政府各项重大制度。健全科学决策机制。完善重大问题集体决策制度、专家咨询制度、社会公示与社会听证制度和决策责任制度，实施政务信息公开办法，使政府决策处于社会和群众监督之下。坚持责任追究制度。实行谁决策谁负责，对超越权限和违反法定程序造成重大损失的决策行为，严肃追究决策者责任，做到有权必有责、用权受监督、侵权须赔偿、违法要追究。严格廉政责任制度。从体制、机制、法制三位一体上推进反腐倡廉工作，加大从源头上预防和治理腐败，建立健全与市场经济体制相适应的教育、制度、监督并重的惩治和预防腐败体系，重点抓好领导干部廉洁自律、查办大案要案和纠正部门行业不正之风三项重点任务。发挥审计监督作用。严格加强土地批租、工程招标、产权交易、政府采购等关键环节的监管，搞好领导干部经济责任审计。

　　大力推进依法行政。努力把政府法制工作与改革发展紧密结合，注重通过立法工作解决体制机制性重大问题。认真实施《行政许可法》和《全面推进依法行政实施纲要》，严格按照法定权限和程序履行行政职责，大力推进法治政府建设。加强政府法制工作。改进政府立法工作模式。立法工作同转变政府职能、贯彻落实科学发展观相结合，在“少而精”上下工夫，扩大公众参与度，不断提高立法质量，使立法机制更加民主化、科学化，切实杜绝部门利益对立法工作的消极影响。实行规范性文件审查和重大行政许可决定备案制度，建立完善政府规章和规范性文件动态清查机制，对不符合经济社会发展需要以及与上位法规冲突的，及时修订或废止。加强社会管理和公共服务方面的立法步伐，为健全各种社会公共应急机制提供立法支持。推进行政综合执法。加强执法协调，建立职能部门与综合执法机构的协调联动机制。认真执行行政复议法。落实行政执法责任制、错案责任追究制和行政赔偿制度，加强行政执法监督，依法维护群众切身利益。

　　着力优化发展环境。努力解决影响发展环境的突出问题，进一步完善优化发展环境的长效机制。继续深化审批制度改革。按照“合法、合理、效能、责任、监督”五项原则和授权办理的要求，进一步精简行政审批事项，简化审批程序，减少办事环节，改进前置性审批，推行“一站式”办公和网上审批，实行“一家受理、转告相关、限时完成、信息共享”的主办部门责任制，大力提高办事效率。集中整治“三乱”问题。以解决扰乱市场经济秩序和损害群众利益的突出问题为重点，深入开展纠风专项治理。健全监督制约机制。继续深入开展“优化发展环境民主评议”活动，建立社会和企业评政府长效机制，强化发展环境投诉中心职能，健全投诉受理快速反应机制，切实解决政府部门工作人员不作为、乱作为、效率低、服务差、影响坏等消极现象，特别要及时解决企业投诉问题，集中力量查处一批损害济南形象的重大案件。加快电子政务建设。大力开展网上查询、网上办事、网上监督等应用服务，以信息化手段实现跨部门信息资源共享和应用，提高政府工作透明度和效率，促进政府运行体制和工作机制的创新。

　　大兴求真务实作风。坚持一切从实际出发。牢固树立科学发展观和正确政绩观，坚决克服官僚主义和形式主义，坚决防止急功近利和短期行为，坚决反对弄虚作假、做表面文章，推行科学的政府绩效评价机制。坚持把握全局突破难点。抓住事关经济社会发展中的突出矛盾、事关群众切身利益的突出问题，勤于调查研究，勇于开拓创新，集中力量加以突破。特别是抓住结构性、深层次矛盾，远有战略目标，近有阶段任务，大力推进经济结构调整，着力加强经济薄弱环节。坚持不遗余力狠抓工作落实。建立严格的工作目标责任制，把经济社会发展的各项目标任务细化、量化，层层分解，逐级落实，抓重点、抓关键、抓调度、抓考核，按照破题到位、策划到位、责任到位、服务到位的要求，确保各项工作落到实处。坚持脚踏实地为民办实事。到最困难的地方去，到群众意见最多的地方去，到工作推不开的地方去，摸实情、办实事、解难题，打开工作局面，特别是重点解决征地补偿安置中侵害农民利益、城镇拆迁中侵害居民利益、企业改制和破产重组中损害职工利益、工程建设中拖欠克扣农民工工资等突出问题，全力维护群众利益。坚持不懈地加强公务员队伍建设。以提高执政意识和行政能力为重点，开展经常性、正规化的培训教育，全面提高综合素质，营造想干事、会干事、干成事的创业氛围，努力建立一支适应新的历史任务要求、人民群众满意的公务员队伍。

　　各位代表，加快推进我市经济和社会发展，是全市人民的共同愿望，是各级政府义不容辞的责任和使命。我们要更加紧密地团结在以胡锦涛同志为总书记的党中央周围，在省委、省政府和市委的领导下，适应新形势，把握新机遇，开创新基业，为争取改革开放和社会主义现代化建设的新胜利而努力奋斗！
